# Antioxidant Supplementation Modulates Neutrophil Inflammatory Response to Exercise-Induced Stress

**DOI:** 10.3390/antiox9121242

**Published:** 2020-12-07

**Authors:** Lucrecia Carrera-Quintanar, Lorena Funes, María Herranz-López, Pascual Martínez-Peinado, Sandra Pascual-García, José M Sempere, Marina Boix-Castejón, Alfredo Córdova, Antoni Pons, Vicente Micol, Enrique Roche

**Affiliations:** 1 Food Science Laboratory, Department of Human Reproduction, Growth and Child Development, University of Guadalajara, Guadalajara 44340, Mexico; lucrecia.carrera@gmail.com; 2 Institute of Research, Development and Innovation in Healthcare Biotechnology of Elche (IDiBE), Miguel Hernández University (UMH), Elche, 03202 Alicante, Spain; llfunesg@gmail.com (L.F.); mherranz@umh.es (M.H.-L.); marinaboix@hotmail.com (M.B.-C.); vmicol@umh.es (V.M.); 3 Department of Biotechnology, University of Alicante, 03690 Alicante, Spain; pascual.martinez@ua.es (P.M.-P.); sandra.pascual@ua.es (S.P.-G.); josemiguel@ua.es (J.M.S.); 4 Department of Biochemistry and Physiology, Faculty of Health Sciences, Soria Campus, University of Valladolid, 42001 Soria, Spain; a.cordova@uva.es; 5 Research Group on Community Nutrition and Oxidative Stress, University of Balearic Islands, 07122 Palma de Mallorca, Balearic Islands, Spain; antonipons@uib.es; 6 CIBER Fisiopatología de la Obesidad y Nutrición (CIBEROBN), Instituto de Salud Carlos III (ISCIII), 28029 Madrid, Spain; 7 Department of Applied Biology-Nutrition and Institute of Bioengineering, Alicante Institute for Health and Biomedical Research (ISABIAL), Miguel Hernández University (UMH), 03010 Alicante, Spain

**Keywords:** antioxidants, inflammation, myeloperoxidase, neutrophils, oxidative stress, polyphenols

## Abstract

The aim of the present report was to evaluate the inflammatory response to a 2000-m running test considering neutrophil myeloperoxidase as an inflammatory marker, and to verify if supplements rich in antioxidants could modulate Post-test antioxidant and anti-inflammatory responses. To this end, a 21-day homogenization period was carried out with three groups: a control group, a supplemented group taking an almond beverage enriched with vitamins C and E and a third group consuming the same beverage but enriched with *Lippia citriodora* extract. At the end of this period, participants performed a 2000-m run, and blood samples were obtained the day before and immediately after the running test. Plasma and neutrophils were isolated. As a result, plasma creatine kinase and myoglobin increased, indicating Post-test muscle damage. Plasma oxidative markers were increased in all groups, except in the group supplemented with the almond beverage. Neutrophil antioxidant enzymes were significantly increased only in the control group, suggesting an antioxidant effect of the supplements provided in the other groups. Myeloperoxidase activity was significantly increased after the test in the control group, while increased enzyme levels were detected in plasma of the supplement groups. Therefore, antioxidant consumption seems to favour myeloperoxidase release. The connection of this observation with post-exercise recovery will require further investigation.

## 1. Introduction

High intensity sports performance is associated with specific stress situations that cause inflammatory and oxidative damage, particularly in the muscle tissue [1]. These include: mechanical stress from repetitive contractile activity, contusions resulting from casual/intentioned contacts and intensified respiratory function in mitochondria [2,3], among others. Subsequently, neutrophils are triggered in response, in a similar fashion as that observed against infections during the acute phase [4]. However, unlike during an infection, the inflammatory and oxidative response is triggered during high intensity exercise in absence of pathogens. The reason for this activation is to activate repair mechanisms of the muscle tissue, favouring an adaptive response to exercise and supporting the subsequent recovery [5,6,7].

The process starts by neutrophil recruitment in response to proteins released from the injured muscle, such as creatine kinase (CK) and myoglobin [8]. Muscle and primed neutrophils release cytokines that attract more neutrophils and macrophages to the damaged area where they are activated [9]. Neutrophil activation requires the assembly of NADPH (nicotinamide adenine dinucleotide phosphate)-oxidase through the fusion of secondary granules to the phagosomal membrane and the generation of reactive oxygen species (ROS) [10]. Neutrophils phagocyte cellular debris, initiating a respiratory burst in which large amounts of superoxide radical (O_2_**^●^**^−^) are produced by the enzyme NADPH-oxidase [11]. Superoxide anions are dismutated by superoxide dismutase (SOD) to form hydrogen peroxide (H_2_O_2_) that is used together with chloride to produce hypochlorous acid (HOCl) and chloramine by myeloperoxidase (MPO) activity [12]. MPO activity depends on the fusion of MPO-containing granules with phagosome [13,14]. In addition, both ROS and HOCl are potent oxidants that cause proteins released from the injured area of the muscle to be phagocyted [15,16]. Inflammatory cytokines released from the damaged muscle and secreted by neutrophils during this process act as extracellular messengers. In particular, interleukins (ILs) 6 and 8 and tumour necrosis factor-α (TNF-α) activate macrophage recruitment that potentiates the phagocytic activity [17]. The process results in muscle debris removal and tissue repair [6,7].

However, ROS and HOCl not only affect the proteins released from the damaged muscle. Other functional plasma proteins and circulating cells undergo collateral oxidation during the process [18]. For this reason, to minimize unspecific damaging effects initiated in the respiratory burst, neutrophils contain an operative system of antioxidants. To complement the activity of intracellular antioxidants, these cells have the ability to efficiently uptake the extracellular antioxidants, such as vitamins C and E [19].

In a previous work [20], we reported that a 2000-m running test can be considered as a valid stress bout for people that are accustomed to train in aerobic routines. We consider that this test is more specific for runners than other ones, such as eccentric repetitions, jumping or downhill running, since it has a strong anaerobic component that causes oxidative and inflammatory stress [21,22]. In addition, endurance races include key moments in which runners change the rhythm and enter the anaerobic threshold. It is likely that in these specific moments, runners are prone to undergo oxidative and inflammatory damage in the muscle tissue. For this reason, runners generally perform this type of training during specific moments of the season. Therefore, the first aim of the present report was to study if neutrophil MPO could give additional information regarding the post-exercise inflammatory process. The second aim was to determine if supplements rich in antioxidants, tested previously in resistance routines [23], could modulate the endogenous antioxidant and anti-inflammatory response after the 2000-m test. There are evidences that dietary supplementation with antioxidants, such as vitamin C and vitamin E, alter the possible anti-inflammatory and antioxidant response to exercise [15,24]. However, the effects of dietary polyphenol supplementation on exercise are still scarce.

## 2. Materials and Methods

### 2.1. Participants and Protocol

All participants were students from the University Miguel Hernandez (Elche, Spain). The participants were randomly assigned to one of the following groups: control group (CG, *n* = 10), supplemented with an almond beverage enriched in vitamins C and E (AB, *n* = 11), and supplemented with the same beverage but enriched with the vitamins and *Lippia citriodora* extract (AB + LE, *n* = 10). The beverage was taken for 22 days, and was previously used in marathon runners in a previous report [25]. Inclusion criteria were to be regular practitioners of aerobic routines, be capable of running 1 km in 4 min maximum time, be free from chronic diseases, non-smokers and follow the nutritional plan prepared by the nutritionist of the research team. Exclusion criteria were to be unable to accomplish the intervention protocol, consumption of sport supplements or prescribed drugs at the moment of the intervention and suffering from recent muscle lesions.

All participants were selected at the beginning of the school year, as it coincides with the end of a long resting vacationing period (summer season). This was made to ensure that all participants were in a similar level of physical activity and to interfere minimally with their subsequent regular training programs in different sport disciplines. Participants were informed about the objective and demands of the intervention and signed the written consent. The study was in accordance with Helsinki Declaration for research on human beings, and approved by the local Ethics Committee with reference IB 544/05 PI.

Before the 2000-m test, we considered a 21-day homogenization period in which participants performed the same aerobic routine and followed a specific diet plan. The aerobic routine consisted in a 90-min race scheduled 90–120 min after breakfast, 3 alternating days a week, during 3 weeks. This results in a total charge of 42–50 km/week monitored by calibrated Polar RS-800 accelerometers (Barcelona, Spain). Diet was designed using Dietsource software (Novartis, Barcelona, Spain) and adapted to aerobic exercises, including 60% carbohydrates, 25% lipids and 15% proteins. Daily energy intakes were estimated in ~2100 kcal for resting days and ~2700 kcal for training days. Energy expenditure was calculated taking into account resting metabolism + thermal effect of food + physical activity expenditure. Resting metabolism was calculated according to the Harris–Benedict equation. The thermal effect of food was estimated as 8.5% of the sum of resting metabolism plus exercise expenditure. Physical activity expenditure was estimated from reference tables. Diet accomplishment was supervised twice a week for each group of participants.

**Figure 1:**
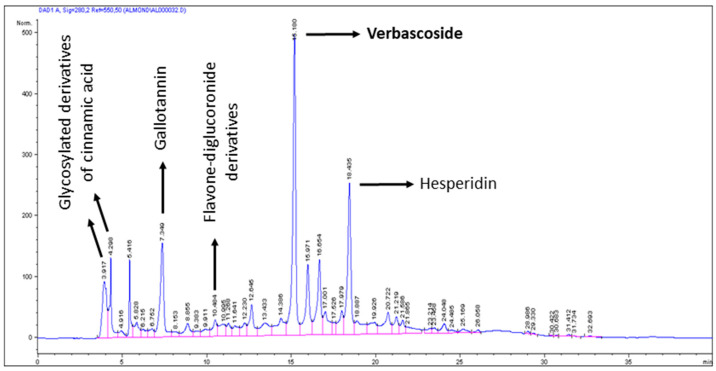
Chromatogram of the almond beverage enriched with *Lippia citriodora.* Hexane extraction was performed twice in 10 mL of the beverage to obtain the aqueous phase. Polyphenols were obtained by methanol:H_2_O: trifluoroacetic acid (70:30:0.3) extraction by incubating 12–16 h, at 4 °C in darkness. Then, samples were centrifuged and supernatant was eliminated by vacuum exposure at 35–40 °C. Pellets were suspended in 5 mL of methanol:H_2_O (1:1) and filtered. In total, 10 µL were analyzed in triplicate by HPLC-DAD-ESI-MS/MS. Verbascoside was present only in *Lippia* extract (see Table 1).

Daily diet vitamin C intakes were ~123 mg for resting days and ~338 mg for training days in all groups. Daily diet vitamin E intakes were ~5 mg for resting days and ~9 mg for training days in all groups. The beverage provided to the AB group consisted in a mixture of crushed almonds and orange juice supplemented with 50 mg of vitamin C/100 mL juice and 20 mg of vitamin E/100 mL juice. The beverage provided to AB + LE group was the same as for AB group, but containing additionally 400 mg of *Lippia citriodora* extract (Monteloeder SL, Elche, Spain). Other components of the beverage were sucrose (0.8%) and fatty acids (12% saturated, 57.5% monounsaturated and 30.5% polyunsaturated) [26]. Participants of both groups consumed 500 mL/day of the corresponding beverages 15–20 min before breakfast to favour absorption of the beverage components. Bioavailability of the *Lippia* extract was extensively studied in animal models [27,28]. The major compounds found in the plasma of rats were the phenylpropanoid verbascoside and isoverbascoside. No data on the bioavailability and pharmacokinetics of these compounds are available from human studies. The beverages were packed by Liquats Vegetals SL (Viladrau, Gerona, Spain) in white cardboard packages displaying only the expiration date that certified the stability of all components during the intervention period. Therefore, the antioxidant potential of the beverage relied on the contents of vitamins C and E and additionally in the presence of polyphenols coming from the crushed almonds and *Lippia* extract, characterized previously by HPLC-MS [27,28,29] (Figure 1) (Table 1). In this context, the beverages were provided to participants with the idea of modulating the endogenous adaptive response, as has been described in detail in a previous report [23].

Recruitment of volunteers was performed among students that regularly compete in various University-level championships. An email was sent to the volunteers, obtaining 45 responses, of which 31 were selected based on anthropometric criteria following ISAK (International Society for Advancement of Kinanthropometry) recommendations [30]. In addition, all participants covered the 2000-m distance in fasting conditions in less than 8 min. The power of the n was 80% for MPO activity and plasma levels with an effect size *d* = 1 and a two-sided 5% significance level.

### 2.2. Blood Sampling and Determination of Circulating Parameters

Blood samples (10–12 mL) were obtained from the antecubital vein after overnight fasting in EDTA vacutainers at the end of the homogenization period (day 20) and 30 min after the 2000-m running test (day 21). Blood extraction at day 20 was performed in fasting conditions at the same hour that the test was scheduled the next day. No training session was performed on that day. Neutrophils and plasma were purified as indicated in [23].

Blood cell counts were determined by an automatic hematology analyzer (Roche Diagnostics, Barcelona, Spain). Circulating glucose, cholesterol, triglycerides, uric acid, urea, creatinine, serum iron, ferritin, lactate and serum Na^+^ and K^+^ were determined as previously described [20]. Plasma proteins, such as CK, myoglobin, alkaline phosphatase, aspartate aminotransferase/serum glutamic oxaloacetic transaminase (AST/GOT), alanine aminotransferase/serum glutamic pyruvic transaminase (ALT/GPT) and γ-glutamyltransferase (GGT) were determined according to standard laboratory procedures as indicated in [23].

### 2.3. Determination of Antioxidant and Myeloperoxidase Enzymatic Activities and Oxidative Stress Markers

Neutrophil catalase (CAT), glutathione peroxidase (GPX), glutathione reductase (GRD) and SOD activities were measured by spectrophotometry on a microplate reader (SPECTROstar Omega, BMG LabTech GmbH, Offenburg, Germany) at 37 °C according to Aebi [31], Flohe and Gunzler [32], Goldberg and Spooner [33] and McCord and Fridovich [34] respectively. Neutrophil MPO activity was determined by guaiacol oxidation as described in [35]. Plasmatic MPO levels were determined by LUMINEX (Austin, TX, USA). Protein carbonyls were determined in neutrophils and plasma in protein precipitates. These were resuspended with 10 mM 2,4-dinitrophenylhydrazine (DNPH) and incubated for 1 h at 37 °C. After incubation, samples were precipitated with 10% trichloroacetic acid (TCA) and centrifuged at 1000× *g* for 10 min at 4 °C. The precipitate was washed twice with ethanol:ethyl acetate (1:1, *v*:*v*) to remove DNPH. Precipitate was resuspended in 6M guanidine in 2 mM phosphate buffer pH 2.3. Samples were incubated for 40 min at 37 °C. At the end of the incubation, samples were centrifuged at 3000× *g* for 5 min at 4 °C to wash the supernatant, and the absorbance was determined at 360 nm. The levels of protein carbonyl derivatives were quantified by using a molar absorption coefficient of 22,000 M^−1^ cm^−1^ and against a blank of guanidine solution. Malondialdehyde (MDA) was determined by HPLC. Neutrophil lysate (100 µL) was mixed with 50 µL of 0.05% butylated hydroxytoluene in ethanol and 50 µL of 20% TCA in 0.6 M HCl. Samples were incubated for 15 min on ice and then centrifuged at 5000× *g* for 15 min at 4 °C. Supernatant (100 µL) were mixed with the same volume of 0.6% thiobarbituric acid (TBA) in water, and the mixture was incubated at 97 °C for 60 min. At the end of incubation, the TBA-MDA chromogen formed was extracted with 300 µL of butanol through vigorous shaking and centrifugation at 10,000× *g* for 3 min. A sample (20 µL) was injected in a reverse phase column LiChrospher^®^ 100-RP-18 (Merck, Darmstadt, Germany) using methanol:50 mM potassium phosphate buffer pH 6.8 (40:60, *v*:*v*) and a flow of 1 mL/min. TBA-MDA chromogen was detected by fluorescence with excitation at 515 nm and emission at 553 nm.

### 2.4. Cytokine Determination

Circulating IL-6, IL-8, IL-10 and TNF-α were determined by Flow Cytometry (FACS-Calibur, BD Bioscience) at the General Hospital of Alicante, using the Flowcytomix^TM^ Multiplex test (eBioscience) (*n* = 3), where 25 μL of plasma was used for IL-8 and TNF-α determination, and 100 μL for IL-6 and IL-10.

### 2.5. Statistical Analysis

Statistical analysis was carried out using the SPSS-26 software for Windows. Data were tested for normality according to the Shapiro–Wilk test. Results were expressed as the mean ± SEM (standard error of the mean). ANOVA test for two factors (supplementation and exercise) was used as statistical analysis. When there was a statistically significant influence of exercise, or supplementation of the interaction between the two factors, the difference between groups was assessed by Student’s *t*-test. Student’s paired *t*-test was used for intragroup analysis at different days and Student’s *t*-test for intergroup analysis at the same time frames. Values with a *p* < 0.05 were considered statistically significant.

## 3. Results

No significant differences were detected among the participants in the different groups with respect to their age, anthropometric parameters and results of the run test (Table 2).

Blood samples obtained the day before the test (day 20 of the training period) in resting conditions from all groups presented circulating parameters (cells and metabolites) in the healthy range, with no significant differences between groups. After 21 days of training, volunteers performed a 2000-m running test and a blood sample was taken 30 min after finishing the test. Lactate, CK and myoglobin increased significantly after the test in all groups compared to basal values (Table 3). The increase in lactate confirmed the anaerobic component during the test. The increases in CK and myoglobin confirmed the presence of muscle damage after the test. In addition, the muscle and liver marker ALT/GPT also increased significantly Post-test in groups AB and AB + LE (Table 3).

Special attention was placed on the neutrophils, as these cells are the first responders to muscle damage. The next step was to verify the presence of an oxidative or inflammatory response due to the muscle damage. The oxidative response was analysed by determining the presence of oxidative markers, such as MDA and protein carbonyls in neutrophils and plasma (Table 4), and the activity of antioxidant enzymes in neutrophils (Table 5). In CG, no changes in neutrophils were detected, and only a significant increase in plasma protein carbonyls was detected after the run. In the AB group, no significant changes regarding oxidative markers were detected in neutrophils and plasma (Table 4). Regarding AB + LE group, only a significant increase in plasma protein carbonyls was observed after the 2000-m test (Table 4). The comparison between groups revealed no significant differences in either of the two markers.

The absence of oxidative stress markers in neutrophils indicated the presence of an adequate antioxidant response. Therefore, we decided to assess if this was due to the effect of endogenous antioxidant enzymes or of the exogenous antioxidants consumed by the participants through the beverages. In the non-supplemented group (CG), antioxidant enzymes SOD and GPX were significantly activated after the 2000-m test compared to the activities determined in resting conditions in neutrophils (Table 5). Post-test catalase and GRD activities did not present significant increases but showed a tendency to increase (Table 5). Altogether, these results confirmed previous results [23]. However, no significant changes were observed for the same antioxidant enzyme activities in AB and AB + LE groups after the test. In addition, SOD and GRD activities in AB and AB + LE groups were significantly lower in the post-run test compared to CG (Table 5). In a previous report [23], we observed that the almond beverage enriched with the *Lippia* extract reduced the expression of genes coding for SOD and GRD. For AB and AB + LE, the results suggested that the antioxidant defences seemed to be exerted by the plasma metabolites derived from the compounds present in the corresponding supplements, complementing the effects of the endogenous antioxidant enzymes.

The other component linked to muscle damage is the inflammatory response. This occurs as a consequence of neutrophil and macrophage infiltration into the damaged area and the release of cytokines acting as extracellular messengers [6]. No significant changes were observed in the levels of IL-6, IL-8, IL-10 and TNF-α in any of the studied groups (Table 6).

In any case, we could not confirm the presence of inflammation after the run, as the cytokines remained the same as base levels. Similarly, the almond beverage enriched with vitamins C and E, and/or with *Lippia* extract, did not seem to modify the post-exercise cytokine profile. To this end, MPO activity was determined in neutrophils and plasma. MPO is an enzyme directly involved in the oxidative-inflammatory burst that is activated after muscle damage [7]. Table 6 reveals that MPO activity in neutrophils was significantly increased after the test in the CG as a result of exercise performance (Table 6). Moreover, post-exercise MPO activity was significantly lower in the supplemented groups compared to CG (Table 6). The changes in MPO activity could reflect the amount of enzyme that pass into plasma during neutrophil activation. Therefore, MPO levels were determined in plasma as well. As a result, CG did not present a significant MPO release in the Post-test situation. Conversely, the supplemented groups presented significant increases in plasmatic MPO compared to the basal situation and to the CG (Table 7).

## 4. Discussion

The present study analyses the oxidative and inflammatory status of University-level athletes after a 2000-m running test, while taking an antioxidant-enriched beverage containing various dietary supplements. All participants performed a similar training program during the homogenization period and followed a similar diet plan. A more complete intervention might consider performing a 2000-m test at the beginning of the homogenization period (day 1). However, very few participants covered 1 km in less than 4 min and blood was not extracted. After completing the training program during the 21-day homogenization period, the majority of the participants were capable of covering the distance in the programmed time. We understand that this could be a limitation of the study that we should take into account in future research.

Certain stress situations that appear during intense sport performance are accompanied by increases in oxidative stress and inflammatory responses [1]. In a previous report [20], it was revealed that a 2000-m running test could be considered as a valid method to assess oxidative stress in individuals that are accustomed to train in aerobic routines. At the end of the test, the participants presented increased oxidative stress and muscle damage [20], confirming the results observed in other studies [21,22]. Conversely, in the AB group, no significant changes regarding oxidative markers were detected in neutrophils and plasma, confirming previous published data [25]. Regarding the AB + LE group, only a significant increase in plasma protein carbonyls was observed after the 2000-m test, confirming previous results with resistance routines [23]. Further studies are necessary in order to assess the mechanisms by which certain antioxidants (i.e., the almond beverage enriched with *Lippia* extract) were less effective against oxidative stress than the components in isolation.

However, in the present study, no significant differences in the expression of the studied cytokines were detected related to exercise performance after the 2000-m test. A similar result was observed in a previous study [20]. This could be due to the extremely short half-life of the messengers in plasma, or that their release did not coincide with the moment that the blood sample was extracted. Despite the fact that there was evidence of an antioxidant response, the same was not clear based on the inflammatory profile. In this context, IL-6 and IL-8 were produced during muscle contraction and they appeared in plasma according to duration and intensity of exercise [36]. The plasmatic presence of IL-10 and TNF-α was delayed compared to IL-6 secretion. TNF-α is associated with muscle damage and seems to be a key signal to start regeneration processes [37]. In addition, the plasma levels of these cytokines seem to be modulated by a variety of factors. However, when inflammation is induced by a potent pro-inflammatory agent, such as lipopolysaccharide, the cytokine response seems to be more consistent [35]. To this end, other parameters such as MPO were analysed, in order to monitor the changes in the inflammatory response after an exercise bout.

Since neutrophil activation is a key part of the inflammatory response, we decided to focus on the information obtained from this cell type. As a result, increased levels of muscle proteins CK and myoglobin were detected as a consequence of the intense exercise (Table 3), which act as signals to elicit muscle repair. Since both oxidation and inflammation were present, the modulation of both parameters was analysed using supplements that contained certain antioxidants (vitamins C, E and polyphenols) as well as anti-inflammatory (polyphenols) compounds [38]. In addition, both supplements have been extensively studied in previous reports [23,24,25,35,39].

The antioxidant response is activated during the process to protect against exercise-induced ROS. This adaptive response could be partially blocked due to an excessive intake of antioxidant supplements [23]. In CG, which did not take any supplement, antioxidant enzymes were activated after the 2000-m test, particularly SOD and GPX. This could explain why neutrophils were protected from oxidative damage after the test, showing no increases in MDA and protein carbonyls (Table 4). Nevertheless, an increase in protein carbonyls was observed but only in plasma, similar to a previous report [20]. We hypothesized that lipid and protein oxidation is easiest controlled in the intracellular neutrophil compartment than in plasma, due to a direct access of antioxidant enzymes to damaged molecules.

In the AB group, the antioxidant and anti-inflammatory potential of the beverage was well characterized previously in endurance practitioners [25]. On the other hand, this report documented that the activity of antioxidant enzymes increased after the marathon in volunteers consuming the same almond beverage [25]. However, the characteristics of the marathon differed from the test performed in the present study. A possible interpretation could be that the antioxidants present in the beverage were capable of controlling the amount of ROS produced immediately after the 2000-m test. Nevertheless, ROS production increased dramatically after a marathon race, and the effect of the antioxidants from the beverage required to be complemented with the endogenous activities. This could be achieved because the almond beverage alone did not interfere with the expression of genes coding for the antioxidant enzymes Cu-Zn-SOD, GPX and GRD [23].

The group AB + LE consumed the same beverage that group AB, but supplemented with *Lippia* extract. This mixture included an excess of polyphenolic antioxidants, which could interfere with antioxidant activities and reduce the gene expression of specific enzymes, particularly Cu-Zn-SOD, Mn-SOD and GRD in neutrophils [23]. In the CG, all antioxidant enzyme activities increased significantly or displayed a tendency to increase (Table 5). On the other hand, same tendency was observed in the AB group except for the glutathione-dependent enzymes (Table 5). However, in the AB + LE group, the GPX and GRD activities increased with respect to the basal situation and compared to the AB group. In a previous publication [40], it was shown in vitro that the polyphenol verbascoside from *Lippia* extract protected the glutathione-dependent enzyme inactivation from oxidation. This could be a possible explanation to the observed results. Nevertheless, additional experiments are necessary to confirm this hypothesis in vivo. Even though not all antioxidant activities increased after the run in AB and AB + LE groups, this did not translate to an increase in neutrophil oxidative markers (Table 4). Our hypothesis is that neutrophils were protected during the post-run test against oxidative damage in both supplemented groups by the combined effect of intracellular antioxidant enzymes and the extracellular antioxidants coming from the supplements. Additional research is necessary to verify this point. In favour of our interpretation, it has been reported that neutrophils have a great capacity to incorporate exogenous antioxidants, including vitamins C and E [19,25,41,42].

MPO is a key enzyme, completing the antimicrobial effect initiated by NADPH-oxidase. However, in the present study that was not infection observed in the participants. Therefore, it is likely that MPO activation in the neutrophils was a consequence of the oxidation of the cell debris produced after exercise, favouring cytokine release and recruiting macrophages [12]. This could be a likely interpretation for results in the CG, where MPO activity increased significantly in circulating neutrophils after the run test (Table 7). On the other hand, MPO levels increased significantly in plasma after the test in the supplemented groups. Our working hypothesis is that dietary supplements could favour MPO release, explaining the decreased activity determined in circulating neutrophils parallel to increased MPO levels in plasma (Table 7). It remains to be verified if all antioxidants could favour MPO release, or if this release occurs only under intense physical exercise, such as the 2000-m run performed in the current study. These key points require further investigation. Nevertheless, we have previously evidenced in isolated neutrophils that vitamin C facilitates neutrophil degranulation in response to PMA stimulation [41]. This coincides with the observations in [42], where neutrophils were reported to be very efficient in accumulating vitamin C. The vitamin was released with MPO and protected substrates such as LDL from uncontrolled oxidation by HOCl. In the current study, we evidenced that dietary supplementation with an almond beverage enriched with vitamin C and vitamin E or enriched with vitamin C, vitamin E plus polyphenols facilitated the neutrophil MPO released after exercise. Furthermore, no differences were observed in this response between the dietary supplementation with the two almond beverages, putting in evidence that an excess of antioxidants did not enhance neutrophil MPO release. A new intervention using only *Lippia* extract supplementation is ongoing in order to see if antioxidant components from the extract could promote MPO release.

Finally, the cause for MPO release in response to exercise is still poorly studied. MPO produces hypochlorite from hydrogen peroxide and chloride, being cytotoxic for cells. However, our hypothesis is that this oxidative burst could be controlled by endogenous antioxidant enzymes whose activity could be modulated by several factors, i.e., testosterone level [43]. In this context, we have previously published that catalase can be released by neutrophils after exercise [44]. Therefore, it could be suggested that the release of antioxidants may be considered a mechanism to control the oxidative damage in which MPO and other oxidative agents are involved. In addition, MPO release may be a strategy to avoid damaging effects after exercise in neutrophils and to accelerate macrophage recruitment for muscle repair, possibly due to MPO becoming inactive in circulation due to the effect of the antioxidants [12,44]. In this vein, activated macrophages can be protected from this pro-oxidant burst by also producing antioxidants, such as 7,8-dihydroneopterin. This compound is an antioxidant generated by macrophages in response to interferon-γ, allowing a more efficient muscle repair [45]. However, this interpretation is only valid for the AB group that presents low oxidative damage (Table 4) together with significant MPO release (Table 7) compared to controls. However, the AB + LE group still display some oxidative damage (Table 4) with a similar MPO release (Table 7) than AB group. We hypothesize that the excess of antioxidants consumed by the AB + LE group may interfere with the protection against MPO. Nevertheless, this proposed mechanism should be addressed in future research in order to decipher the exact role of antioxidants (amount and type) in controlling the pro-oxidant activity and release of MPO during post-exercise recovery.

## 5. Conclusions

Neutrophils were protected from oxidative damage post-exercise by the activation of their own antioxidant enzymes. In this scenario, antioxidant supplements can protect neutrophils against oxidative damage post-exercise with no significant activation of intracellular enzymes. In addition, neutrophil MPO release is favoured by antioxidant supplements.

## Figures and Tables

**Table 1:** Concentrations of the main peaks indicated in Figure 1. Results expressed in mg/L of almond beverage without and with *Lippia* extract after duplicate polyphenol extraction and triplicate HPLC-DAD-ESI-MS/MS analysis.

Supplement	Glycosylated Derivatives of Cinnamic Acid	Gallotannin	Flavone-Diglucuronide Derivatives	Verbascoside	Hesperidin
Almond beverage	4.3 ± 0.4	62.8 ± 10	3.2 ± 0.0	-	60.4 ± 5
Almond beverage enriched with *Lippia* extract	2.6 ± 0.1	32.7 ± 2	3.9 ± 0.2	90 ± 0.6	46 ± 3

**Table 2:** Age, anthropometric parameters (determined at the moment of recruitment) and results of the 2000-m test in control (CG), almond beverage (AB) and almond beverage + *Lippia* extract (AB + LE) groups.

Parameter (Units)	CG	AB	AB + LE
N	10	11	10
Age (years)	22 ± 1.5	21 ± 1.2	22 ± 0.5
Height (cm)	173 ± 2	174 ± 2	175 ± 3
Weight (kg)	71.8 ± 2.6	70.5 ± 2.0	72 ± 0.9
BMI (kg/m^2^)	22.2 ± 0.9	23.5 ± 1.1	24.0 ± 1.3
Fat mass (%)	14.6 ± 1.3	14.0 ± 1.3	14.3 ± 1.2
Muscle mass (%)	42.0 ± 2.7	44.4 ± 2.3	41.5 ± 2.5
Test time (min)	7.9 ± 0.3	7.8 ± 0.3	8.0 ± 0.2

Abbreviations used: BMI, body mass index.

**Table 3:** Lactate and serum parameters related to muscle damage determined the day before the test (basal) and immediately after the 2000-m test (post-exercise) in control (CG), almond beverage (AB) and almond beverage + *Lippia* extract (AB + LE) groups.

Parameter (Units)	CG	AB	AB + LE
**Basal**			
Lactate (mg/dL)	8.5 ± 0.5	8.8 ± 0.6	7.9 ± 0.7
Myoglobin (ng/mL)	39.5 ± 3.0	34.4 ± 3.7	35.2 ± 4.1
GGT (U/L)	17.2 ± 0.8	17.1 ± 1.0	16.4 ± 1.0
AST/GOT (U/L)	23.3 ± 0.8	22.6 ± 1.3	23.8 ± 1.0
ALT/GPT (U/L)	19.8 ± 1.3	20.3 ± 1.3	19.3 ± 1.2
CK (U/L)	212 ± 18	195 ± 22	195 ± 12
Alkaline phosphatase (U/L)	186 ± 17	154 ± 9	173 ± 19
**Post-exercise**			
Lactate (mg/dL)	11.6 ± 2.5 *	12.2 ± 2.7 *	11.0 ± 1.3 *
Myoglobin (ng/mL)	45.2 ± 3.8 *	45.7 ± 2.9 *	39.9 ± 2.3 *
GGT (U/L)	18.4 ± 1.7	17.4 ± 0.9	18.1 ± 0.9
AST/GOT (U/L)	25.8 ± 1.6	24.3 ± 0.9	25.1 ± 1.2
ALT/GPT (U/L)	21.3 ± 1.2	26.4 ± 1.7 *	28.8 ± 1.3 *
CK (U/L)	275 ± 23 *	268 ± 30 *	264 ± 26 *
Alkaline phosphatase (U/L)	173 ± 19	166 ± 12	186 ± 17

* Significant differences (*p* < 0.05) after 2000-m test performance compared to basal conditions in the same group. Abbreviations used: AST/GOT aspartate aminotransferase/serum glutamic oxaloacetic transaminase, ALT/GPT alanine aminotransferase/serum glutamic pyruvic transaminase, CK creatine kinase, GGT γ-glutamyltransferase.

**Table 4:** Presence of oxidative markers in neutrophils and plasma determined in basal conditions and immediately after the 2000-m test in control (CG), almond beverage (AB) and almond beverage + *Lippia* extract (AB + LE) groups.

Marker (Units)		CG	AB	AB + LE	ANOVA
**Neutrophils**					
MDA (mmols/L)	Basal	7.1 ± 2.1	7.6 ± 1.8	6.9 ± 1.9	
	Post-exercise	6.5 ± 1.1	8.2 ± 2.8	7.2 ± 1.2	
Protein carbonyls (mmols/L)	Basal	11.6 ± 2.1	10.6 ± 1.4	11.1 ± 1.7	
	Post-exercise	12.4 ± 2.5	10.6 ±1.4	12.6 ± 2.3	
**Plasma**					
MDA (µmols/L)	Basal	2.6 ±1.3	2.8 ± 0.5	3.4 ± 1.3	
	Post-exercise	2.6 ± 1.4	3.0 ± 1.3	2.8 ± 0.3	
Protein carbonyls (µmols/L)	Basal	80.4 ± 2.3	86.0 ± 7.9	85.8 ± 9.4	E
	Post-exercise	95.8 ± 7.4 *	91.3 ± 8.9	104 ± 10 *	

* Significant differences (*p* < 0.05) due to exercise (E) according to ANOVA test compared to basal conditions in the same group. Intracellular malondialdehyde (MDA) and protein carbonyl concentrations were calculated assuming a value of 300 µL/10^9^ cells [30].

**Table 5:** Antioxidant enzymatic activities determined in neutrophils in basal conditions and immediately after the 2000-m test in control (CG), almond beverage (AB) and almond beverage + *Lippia* extract (AB + LE) groups.

Enzymatic Activity		CG	AB	AB + LE	ANOVA
SOD (pkat/10^9^ cells)	Basal	26.6 ± 6.7	20.3 ± 4.3	23.0 ± 2.7	E SxE
	Post-Exercise	47.5 ± 7.0 *	27.6 ± 6.8 *^&^	21.3 ± 4.7 *^&^	
CAT (k_5_/10^9^ cells)	Basal	58.2 ± 16.7	56.2 ± 11.7	60.0 ± 15.1	S
	Post-Exercise	70.8 ±12.2	82.2 ± 19.7	116 ± 39 ^&^	
GPX (nkat/10^9^ cells)	Basal	78.9 ± 20.1	85.2 ± 28.4	88.7 ± 24.3	E SxE
	Post-Exercise	127 ± 11 *	76.6 ± 7.7 *^&^	146 ± 42	
GRD (nkat/10^9^ cells)	Basal	369 ± 103	376 ± 92	317 ± 53	S
	Post-Exercise	442 ± 47	316 ± 36 ^&^	351 ± 36 ^&^	

* Significant differences (*p* < 0.05) due to exercise (E), ^&^ significant differences (*p* < 0.05) due to supplement consumption (S) or *^&^ significant differences (*p* < 0.05) due to an interaction between both factors (SxE), according to ANOVA test. Abbreviations used: CAT, catalase; GPX, glutathione peroxidase; GRD, glutathione reductase; SOD, superoxide dismutase.

**Table 6:** Plasma cytokine levels determined in basal conditions and immediately after the 2000-m test in control (CG), almond beverage (AB) and almond beverage + *Lippia* extract (AB + LE) groups.

Cytokine (Units)		CG	AB	AB + LE
IL-6 (pg/mL)	Basal	1.6 ± 0.7	1.3 ± 0.3	2.4 ± 1.3
	Post-Exercise	1.8 ± 0.7	1.9 ± 0.7	1.8 ± 0.9
IL-8 (pg/mL)	Basal	6.2 ± 1.7	4.2 ± 1.6	2.9 ± 1.4
	Post-test	6.3 ±2.0	5.9 ± 1.7	5.5 ± 1.6
IL-10 (fg/mL)	Basal	339 ± 91	365 ± 98	282 ± 96
	Post-test	320 ± 125	324 ± 123	309 ± 94
TNF-α (pg/mL)	Basal	8.7 ± 3.2	6.0 ± 2.4	7.1 ± 3.5
	Post-test	5.3 ± 3.6	6.6 ± 2.4	7.9 ± 1.4

No significant changes were observed.

**Table 7:** MPO enzymatic activity in neutrophils and plasma levels determined in basal conditions and immediately after the 2000-m test in control (CG), almond beverage (AB) and almond beverage + *Lippia* extract (AB + LE) groups.

		CG	AB	AB + LE	ANOVA
**Neutrophils**					
MPO Activity (µkat/10^9^cells)	Basal	38.6 ± 7.9	35.1 ± 2.8	35.4 ± 2.9	**E SxE**
	Post-Exercise	59.5 ± 9.6 *	39.5 ± 4.4 *^&^	35.8 ± 3.3 *^&^	
**Plasma**					
MPO levels (ng/mL)	Basal	38.5 ± 8.2	41.2 ± 3.3	48.7 ± 6.7	**SxE**
	Post-Exercise	40.1 ± 4.5	49.5 ± 4.0 *^&^	55.6 ± 3.3 *^&^	

* Significant differences (*p* < 0.05) due to exercise (E) or *^&^ significant differences (*p* < 0.05) due to an interaction between supplement and exercise (SxE), according to ANOVA test. Abbreviations used: MPO, myeloperoxidase.

## References

[1] Nieman D.C., Mitmesser S.H. (2017). Potential impact of nutrition on immune system recovery from heavy exertion: A metabolomics perspective. Nutrients.

[2] McGinley C., Shafat A., Donnelly A.E. (2009). Does antioxidant vitamin supplementation protect against muscle damage?. Sports Med..

[3] Morton J.P., Kayani A.C., McArdle A., Drust B. (2009). The exercise induced stress response of skeletal muscle, with specific emphasis on humans. Sports Med..

[4] Niess A.M., Simon P. (2007). Response and adaptation of skeletal muscle to exercise: The role of reactive oxygen species. Front. Biosci..

[5] Pattwell D.M., Jackson M.J. (2004). Contraction-induced oxidants as mediators of adaptation and damage in skeletal muscle. Exerc. Sport Sci. Rev..

[6] Tidball J.G. (2005). Inflammatory processes in muscle injury and repair. Am. J. Physiol. Regul. Integr. Comp. Physiol..

[7] Toumi H., F’Guyer S., Best T.M. (2006). The role of neutrophils in injury and repair following muscle stretch. J. Anat..

[8] Brancaccio P., Lippi G., Maffulli N. (2010). Biochemical markers of muscle damage. Clin. Chem. Lab. Med..

[9] Lee E.C., Fragala M.S., Kavouras S.A., Queen R.M., Pryor J.L., Casa D.J. (2017). Biomarkers in sports and exercise: Tracking health, performance, and recovery in athletes. J. Strength Cond. Res..

[10] Jesaitis A.J., Buescher E.S., Harrison D., Quinn M.T., Parkos C.A., Livesey S., Linner J. (1990). Ultrastructural localization of cytochrome b in the membranes of resting and phagocytosing human granulocytes. J. Clin. Investig..

[11] Quinn M.T., Gauss K.A. (2004). Structure and regulation of neutrophil respiratory burst oxidase: Comparison with non-phagocyte oxidases. J. Leukoc. Biol..

[12] Khan A.A., Alsahli M.A., Rahmani A.H. (2018). Myeloperoxidase as an active disease biomarker: Recent biochemical and pathological perspectives. Med. Sci..

[13] Bhattacharya A., Wei Q., Shin J.N., Fattah E.A., Bonilla D.L., Xiang Q., Eissa N.T. (2015). Autophagy is required for neutrophil-mediated inflammation. Cell Rep..

[14] Morozov V.I., Pryatkin S.A., Kalinski M.I., Rogozkin V.A. (2003). Effect of exercise to exhaustion on myeloperoxidase and lysozyme release from blood neutrophils. Eur. J. Appl. Physiol..

[15] Clarkson P.M., Thompson H.S. (2000). Antioxidants: What role do they play in physical activity and health?. Am. J. Clin. Nutr..

[16] Moylan J.S., Reid M.B. (2007). Oxidative stress, chronic disease, and muscle wasting. Muscle Nerve.

[17] Kanda K., Sugama K., Hayashida H., Sakuma J., Kawakami Y., Miura S., Yoshioka H., Mori Y., Suzuki K. (2013). Eccentric exercise-induced delayed-onset muscle soreness and changes in markers of muscle damage and inflammation. Exerc. Immunol. Rev..

[18] Zeng M.Y., Miralda I., Armstrong C.L., Uriarte S.M., Bagaitkar J. (2019). The roles of NADPH oxidase in modulating neutrophil effector responses. Mol. Oral. Microbiol..

[19] Tauler P., Aguiló A., Fuentespina E., Tur J.A., Pons A. (2002). Diet supplementation with vitamin E, vitamin C and beta-carotene cocktail enhances basal neutrophil antioxidant enzymes in athletes. Pflug. Arch..

[20] Carrera-Quintanar L., Funes L., Sánchez-Martos M., Martínez-Peinado P., Sempere J.M., Pons A., Micol V., Roche E. (2017). Effect of a 2000-m running test on antioxidant and cytokine response in plasma and circulating cells. J. Physiol. Biochem..

[21] Bloomer R.J., Fry A.C., Falvo M.J., Moore C.A. (2007). Protein carbonyls are acutely elevated following single set anaerobic exercise in resistance trained men. J. Sci. Med. Sport.

[22] Groussard C., Rannou-Bekono F., Machefer G., Chevanne M., Vincent S., Sergent O., Cillard J., Gratas-Delamarche A. (2003). Changes in blood lipid peroxidation markers and antioxidants after a single sprint anaerobic exercise. Eur. J. Appl. Physiol..

[23] Carrera-Quintanar L., Funes L., Vicente-Salar N., Blasco-Lafarga C., Pons A., Micol V., Roche E. (2015). Effect of polyphenol supplements on redox status of blood cells: A randomized controlled exercise training trial. Eur. J. Nutr..

[24] Cases N., Aguiló A., Tauler P., Sureda A., Llompart I., Pons A., Tur J.A. (2005). Differential response of plasma and immune cell’s vitamin E levels to physical activity and antioxidant vitamin supplementation. Eur. J. Clin. Nutr..

[25] Sureda A., Tauler P., Aguiló A., Cases N., Llompart I., Tur J.A., Pons A. (2007). Antioxidant supplementation influences the neutrophil tocopherol associated protein expression, but not the inflammatory response to exercise. Cent. Eur. J. Biol..

[26] Martorell M., Capó X., Sureda A., Batle J.M., Llompart I., Argelich E., Tur J.A., Pons A. (2014). Effect of DHA on plasma fatty acid availability and oxidative stressduring training season and football exercise. Food Funct..

[27] Quirantes-Piné R., Herranz-López M., Funes L., Borrás-Linares I., Micol V., Segura-Carretero A., Fernández-Gutiérrez A. (2013). Phenylpropanoids and their metabolites are the major compounds responsible for blood-cell protection against oxidative stress after administration of Lippia citriodora in rats. Phytomedicine.

[28] Funes L., Fernandez-Arroyo S., Laporta O., Pons A., Roche E., Segura-Carretero A., Fernandez-Gutierrez A., Micol V. (2009). Correlation between plasma antioxidant capacity and verbascoside levels in rats after oral administration of lemon verbena extracts. Food Chem..

[29] Mestre-Alfaro A., Ferrer M.D., Sureda A., Tauler P., Martinez E., Bibiloni M.M., Micol V., Tur J.A., Pons A. (2011). Phytoestrogens enhance antioxidant enzymes after swimming exercise and modulate sex hormone plasma levels in female swimmers. Eur. J. Appl. Physiol..

[30] Marfell-Jones M., Olds T., Steward A., Carter L. (2006). International Standards for Anthropometric Assessment.

[31] Aebi H. (1984). Catalase in vitro. Methods Enzymol..

[32] Flohe L., Gunzler W.A. (1984). Assays of glutathione peroxidase. Methos Enzymol..

[33] Goldberg D.M., Spooner R.J., Bergmeyer H.U. (1985). Glutathione reductase. Methods in Enzymatic Analysis.

[34] McCord J.M., Fridovich I. (1969). Superoxide dismutase. An enzymic function for erythrocuprein (hemocuprein). J. Biol. Chem..

[35] Funes L., Carrera-Quintanar L., Cerdán-Calero M., Ferrer M.D., Drobnic F., Pons A., Roche E., Micol V. (2011). Effect of lemon verbena supplementation on muscular damage markers, proinflammatory cytokines release and neutrophils’ oxidative stress in chronic exercise. Eur. J. Appl. Physiol..

[36] Febbraio M.A., Steensberg A., Keller C., Starkie R.L., Nielsen H.B., Krustrup P., Ott P., Secher N.H., Pedersen B.K. (2003). Glucose ingestion attenuates interleukin-6 release from contracting skeletal muscle in humans. J. Physiol..

[37] Pedersen B.K. (2013). Muscle as a secretory organ. Compr. Physiol..

[38] Hussain T., Tan B., Yin Y., Blachier F., Tossou M.C., Rahu N. (2016). Oxidative stress and inflammation: What polyphenols can do for us?. Oxid. Med. Cell. Longev..

[39] Martínez-Rodríguez A., Moya M., Vicente-Salar N., Brouzet T., Carrera-Quintanar L., Cervelló E., Micol V., Roche E. (2015). Biochemical and psychological changes in university students performing aerobic exercise and consuming lemon verbena extracts. Curr. Top. Nutraceut. Res..

[40] Carrera-Quintanar L., Funes L., Viudes E., Tur J., Micol V., Roche E., Pons A. (2012). Antioxidant effect of lemon verbena extracts in lymphocytes of university students performing aerobic training program. Scand. J. Med. Sci. Sports.

[41] Capó X., Martorell M., Sureda A., Tur J.A., Pons A. (2015). Effects of docosahexaenoic supplementation and in vitro vitamin C on the oxidative and inflammatory neutrophil response to activation. Oxid. Med. Cell Longev..

[42] Carr A.C., Tijerina T., Frei B. (2000). Vitamin C protects against and reverses specific hypochlorous acid- and chloramine-dependent modifications of low-density lipoprotein. Biochem. J..

[43] Marin D.P., Bolin A.P., dos Santos R.C.M., Curi R., Otton R. (2010). Testosterone suppresses oxidative stress in human neutrophils. Cell Biochem. Funct..

[44] Sureda A., Ferrer M.D., Tauler P., Maestre I., Aguiló A., Córdova A., Tur J.A., Roche E., Pons A. (2007). Intense physical activity enhances neutrophil antioxidant enzyme gene expression. Immunocytochemistry evidence for catalase secretion. Free Rad. Res..

[45] Gieseg S.P., Baxter-Parker G., Lindsay A. (2018). Neopterin, inflammation, and oxidative stress: What could we be missing?. Antioxidans.

